# Draft Genome Sequence of Bivalent *Clostridium botulinum* Strain IBCA10-7060, Encoding Botulinum Neurotoxin B and a New FA Mosaic Type

**DOI:** 10.1128/genomeA.01275-14

**Published:** 2014-12-11

**Authors:** Narjol Gonzalez-Escalona, Nagarajan Thirunavukkarasu, Ajay Singh, Magaly Toro, Eric W. Brown, Donald Zink, Andreas Rummel, Shashi K. Sharma

**Affiliations:** a Division of Microbiology, Office of Regulatory Science, Center for Food Safety and Applied Nutrition, Food and Drug Administration, College Park, Maryland, USA; b Office of the Center Director, Center for Food Safety and Applied Nutrition, Food and Drug Administration, College Park, Maryland, USA; c Institut für Toxikologie, Medizinische Hochschule Hannover, Niedersachsen, Germany

## Abstract

Here we report the genome sequence of a *Clostridium botulinum* strain IBCA10-7060 producing botulinum neurotoxin serotype B and a new toxin serotype. Multilocus sequence typing analysis revealed that this strain belongs to a new sequence type, and whole-genome single nucleotide polymorphism analysis showed that this strain clustered with strains in lineage 2 from group I.

## GENOME ANNOUNCEMENT

*Clostridium botulinum* is a Gram-positive, spore-forming anaerobic bacterium that produces botulinum neurotoxin (BoNT) (1). Intoxication with the potent BoNT causes the serious paralytic illness botulism in humans and is a serious concern for food safety. The neurotoxins produced by these organisms are serologically differentiated into seven serotypes, designated by the letters A through G (2).

*C. botulinum* genomes show tremendous genetic diversity and variations in terms of their genome sequence, the serotype they produce, their toxin gene cluster type and organizations, and their locations in plasmids, chromosomes, and bacteriophages (3–5). Genetic analysis of the toxin serotype gene clusters reveal dynamic recombination and horizontal transfer of toxin genes across various phylogenetic groups of neurotoxigenic clostridia. Hence, genome analysis for toxigenic *C. botulinum* strains is of high significance for epidemiological understanding of their animal and human, including infant, host range relationships; for understanding their adaptive interactions with food and environmental samples; and for investigating outbreaks (6). Previously we established the existence of five lineages within *C. botulinum* group I through whole-genome sequencing, single nucleotide polymorphism (WGS-SNP) analysis, which provided higher strain-specific genome resolution in the phylogenetic analysis even within group/lineage types (6).

To contemplate public health emergency preparedness and response toward botulism outbreaks, the genome of a dual toxin-producing *Clostridium botulinum* strain IBCA10-7060, which causes infant botulism (7), was sequenced. The isolate was sequenced using the MiSeq Illumina version 2 kit (2 × 250 bp) (Illumina, San Diego, CA, USA) according to the manufacturer’s instructions at 140× coverage. Genomic DNA from the strain was isolated from overnight cultures with the DNeasy blood and tissue kit (QIAGEN, Valencia, CA, USA). The libraries were constructed with the Nextera XT kit (Illumina), according to manufacturer’s instructions, using 1 ng of genomic DNA. Genomic sequence contigs for strain IBCA10-7060 were *de novo* assembled using the CLC Genomics Workbench version 7.5 (CLC bio, Germantown, MD, USA). The G+C content of this strain was 28.1%, which is similar to the reported G+C content for other *C. botulinum* strains (6). Sequences were annotated using the NCBI Prokaryotic Genomes Automatic Annotation Pipeline (PGAAP, http://www.ncbi.nlm.nih.gov/genome/annotation_prok).

The genome sequence confirmed that the strain possesses a *bont*/B gene associated with the *ha* gene cluster and the new *bont*/FA mosaic gene sequence associated with the *orf*X gene cluster, as reported earlier (8). Preliminary analysis of the resulting sequence contigs indicated lack of plasmids, as well as any other cryptic neurotoxin genes in this strain. Comparing other genome sequences of strains belonging to the proteolytic group I, we report that IBCA10-7060 belongs to a new sequence type, which is closest to ST55 isolated (277-00) in France in 2000, differing by a single SNP in the *acek* allele. Whole-genome SNP analysis showed that this strain clustered with strains belonging to lineage 2 that are mostly bivalent strains, as inferred from our previous studies (6).

A detailed report of phylogenetic analysis of the draft genome will be included in future publications.

### Nucleotide sequence accession number.

The draft genome sequence of the *C. botulinum* strain is available in GenBank under accession number JSCF00000000.
